# Two *Paenibacillus* spp. strains promote grapevine wood degradation by the fungus *Fomitiporia mediterranea*: from degradation experiments to genome analyses

**DOI:** 10.1038/s41598-024-66620-x

**Published:** 2024-07-09

**Authors:** Rana Haidar, Stéphane Compant, Coralie Robert, Livio Antonielli, Amira Yacoub, Axelle Grélard, Antoine Loquet, Günter Brader, Rémy Guyoneaud, Eléonore Attard, Patrice Rey

**Affiliations:** 1 E2S UPPA, CNRS, IPREM UMR5254, Université de Pau et des Pays de l’Adour, Pau, France; 2 INRAE, UMR1065 Santé et Agroécologie du Vignoble (SAVE), ISVV, 33883 Villenave d’Ornon, France; 3 Bioresources Unit, Center for Health and Bioresources, AIT Austrian Institute of Technology GmbH, Konrad Lorenz Straße 24, 3430 Tulln, Austria; 4 Institut de Chimie et Biologie des Membranes et des Nanoobjets, IECB, CNRS, Université de Bordeaux, 33607 Pessac, France

**Keywords:** Bacteria, Fungi

## Abstract

Ascomycetes, basidiomycetes and deuteromycetes can degrade wood, but less attention has been paid to basidiomycetes involved in Esca, a major Grapevine Trunk Disease. Using a wood sawdust microcosm system, we compared the wood degradation of three grapevine cultivars inoculated with *Fomitiporia mediterranea* M. Fisch, a basidiomycete responsible for white-rot development and involved in Esca disease. The grapevine cultivar Ugni blanc was more susceptible to wood degradation caused by *F. mediterranea* than the cultivars Cabernet Sauvignon and Merlot. Solid-state Nuclear Magnetic Resonance (NMR) spectroscopy showed that *F. mediterranea* preferentially degrades lignin and hemicellulose over cellulose (preferential, successive or sequential white-rot). In addition, co-inoculation of sawdust with two cellulolytic and xylanolytic bacterial strains of *Paenibacillus* (Nakamura) Ash** (***Paenibacillus* sp. (S231-2) and *P. amylolyticus* (S293)), enhanced *F. mediterranea* ability to degrade Ugni blanc. The NMR data further showed that the increase in Ugni blanc sawdust degradation products was greater when bacteria and fungi were inoculated together. We also demonstrated that these two bacterial strains could degrade the wood components of Ugni blanc sawdust. Genome analysis of these bacterial strains revealed numerous genes predicted to be involved in cellulose, hemicellulose, and lignin degradation, as well as several other genes related to bacteria-fungi interactions and endophytism inside the plant. The occurrence of this type of bacteria-fungus interaction could explain, at least in part, why necrosis develops extensively in certain grapevine varieties such as Ugni blanc.

## Introduction

The development of grapevine trunk disease (GTD) has become a subject of utmost importance in international viticulture since the 2000s^1,2^. GTDs are associated with wood degradation and the development of various types of wood necrosis. Generally, studies on microbial wood decomposition have suggested that fungi are the main decomposers because of their ability to produce many enzymes involved in lignin, cellulose, and hemicellulose degradation, which destroy wood biopolymers^3–5^. Pathogenic fungi are usually reported as the key microorganisms that degrade grapevine wood in Esca, which is the most widespread GTD worldwide. They are responsible for the discoloration of wood and the development of white-rot necrosis, a final structure in wood degradation, and the most typical symptom of Esca disease^1,2,6,7^ also called “amadou” in French. *Fomitiporia mediterranea* is the main white-rotting basidiomycete of grapevine in Europe and the Mediterranean regions. This fungus with a high ligninolytic activity, can degrade grapevine wood tissues and is usually involved in Esca disease^8–10^_._

Over the last decade, it has been shown that fungi are not the only microorganisms that colonize grapevine wood tissues, and the occurrence of bacteria in these tissues has been reported in several studies^11–14^. Although certain bacteria could contribute to decay in various plants, including wheat, rice, corn, soybean, and grapevine^15,16^, they have a limited ability to decompose wood components^17,18^. However, it is assumed that the interaction of bacteria with fungi could lead to wood degradation processes^15–21^, and that the synergistic role of bacteria with pathogenic basidiomycetes could increase the degradation of wood components^19,22–24^.

The interactions of bacteria colonizing grapevines with wood-decaying fungi have already been investigated in the context of GTDs. However, most studies have focused on the biocontrol of pathogenic fungi by bacteria^25,26^. Only a few publications^15,27^ have investigated the synergistic interactions between these fungi and wood-inhabiting bacteria. For instance, Haidar et al^27^ observed an increase in the canker development on grapevine stem cuttings after co-inoculating *Bacillus pumilus* strain S35 and *Xanthomonas* sp. strain S45 with *Neofusicoccum parvum,* a GTD fungus. In addition, it was showed that wood degradation by *F. mediterranea* was enhanced in the presence of a *Paenibacillus* strain identified as a new species of bacteria named *Paenibacillus xylinteritus*^28^. Genome annotation of this last strain revealed the presence of several gene clusters related to carbohydrate-active enzymes, xylose degradation and vitamin metabolism.

To decipher putative interactions between *F. mediterranea* and some bacteria, we: (i) compared the susceptibility of three grapevine cultivars (Cabernet Sauvignon, Merlot, and Ugni blanc) to *F. mediterranea*, and (ii) described the ability of two bacterial strains, *P. amylolyticus* strain S293 and *Paenibacillus* sp. strain S231-2, to enhance wood colonization and decomposition by *F. mediterranea*. The cultivar most sensitive to *F. mediterranea* attack, Ugni blanc, was then selected to study the bacteria-fungus interaction. The two bacterial strains were selected from a previous experiment using wood microcosms (i.e., the medium was made of grapevine sawdust), as they displayed strong cellulase and xylanase activities and did not inhibit the growth of *F. mediterranea* mycelia^15^. However, their role in wood degradation was not determined. Finally, whole genome annotation of the two bacterial strains was performed to determine their potential role to degrade wood components and to produce secondary metabolites that could be involved in bacterial-fungal-plant interactions.

## Results

### Difference in the susceptibility of three grapevine cultivars to F. mediterranea attack

#### *F. mediterranea*’s mycelial growth on tested grapevine cultivars wood

**Figure 1:**
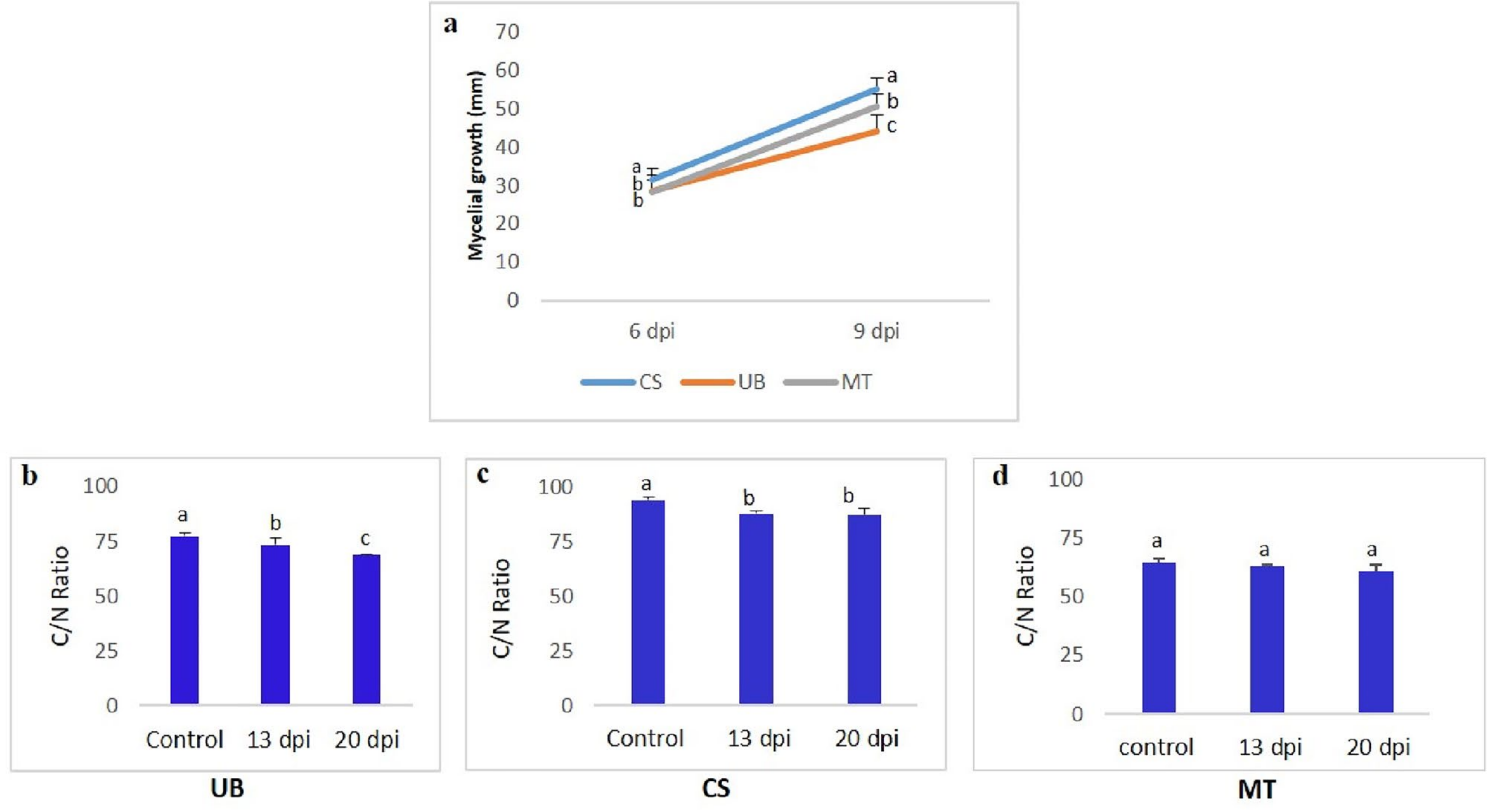
(**a**) Measurement of mycelial growth of *F. mediterranea* on sawdusts of tested cultivars (CS: Cabernet Sauvignon, UB: Ugni blanc, MT: Merlot) at 6 and 9 dpi. (**b–d**) Wood decay characterization after 13 and 20 days of the inoculation of *F. mediterranea* on sawdust of UB and CS. Different letters indicate significantly different at *P* ≤ 0.05, according to the Newman and Keul’s test after ANOVA. The error bar corresponds to the standard deviation of the mean.

Measurement of the mycelial growth of *F. mediterranea* on wood of three cultivars (Cabernet Sauvignon, Merlot, and Ugni blanc) at six and nine days post-inoculation (dpi) revealed that *F. mediterranea* development depended on the grapevine cultivar. As shown in Fig. 1a, *F. mediterranea* developed faster on Cabernet Sauvignon until nine dpi than on the other cultivars. *F. mediterranea* demonstrated the slowest growth on Ugni blanc sawdust (Fig. 1a).

#### Degradation of wood of different grapevine cultivars by *F. mediterranea*

As shown in Fig. S1, the sawdust of all cultivars inoculated with *F. mediterranea* had a lighter color (more yellow) than the control sawdust at 13 and 20 dpi.

Carbon and nitrogen concentrations were measured in all cultivars after 13 and 20 days of incubation. A comparison of the C/N ratio at the three sampling times (T0 (control), T13, and T20) showed that the cultivar significantly affected (*P* = 0.02) wood degradation by *F. mediterranea.*

Contrary to the Merlot sawdust showing no difference in the C/N ratio at 13 and 20 dpi, the C/N ratios of Cabernet Sauvignon and Ugni blanc were significantly higher in sterile sawdust (control) than in other sawdust samples inoculated with *F. mediterranea* after 13 and 20 days of incubation (Fig. 1b–d). Interestingly, between 13 and 20 days of incubation, the C/N ratio significantly decreased in Ugni blanc sawdust, suggesting that *F. mediterranea* degrades the wood of this cultivar more efficiently (Fig. 1b). While the wood loss rates were 30 and 42% after 13 and 20 days, respectively, for Ugni blanc, the same rates were 15 and 19% for CS at 13 and 20 days, respectively.

**Figure 2:**
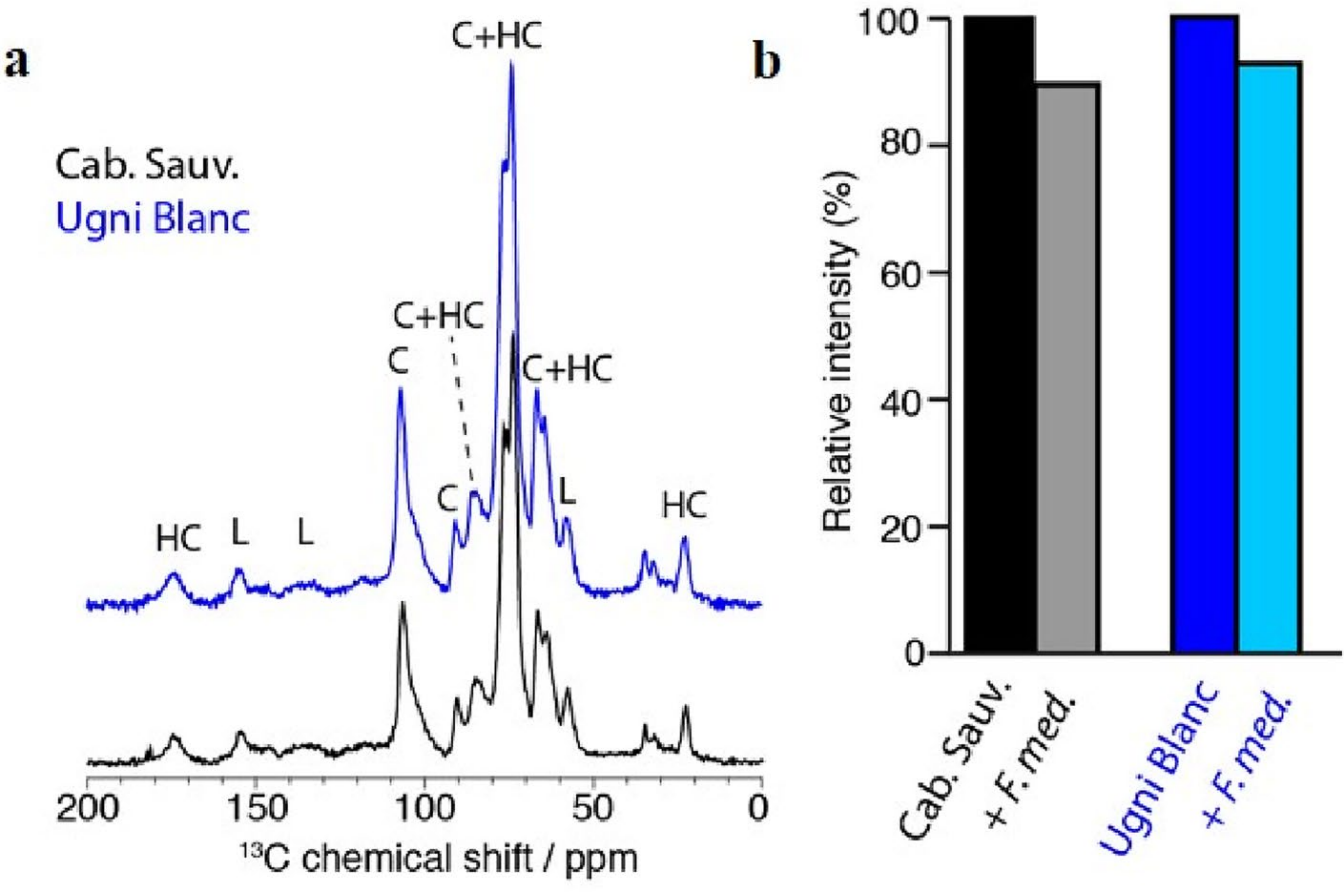
Solid-state NMR wood decay characterization after inoculation of *F. mediterranea* on Cabernet Sauvignon and Ugni blanc sawdusts. (**a**) ^13^C cross-polarization spectra of CS and UB sawdust. (**b**) Total wood decay as measured by NMR intensity.

To corroborate these results, we employed Magic-angle spinning (MAS) solid-state NMR to investigate wood degradation at the molecular level. MAS NMR is a powerful technique^29–31^ for studying the molecular composition of wood, quantifying the structural polymeric components, and investigating wood degradation^15^. To measure the total wood decay, we performed ^13^C-detected cross-polarization experiments on control wood samples of Cabernet Sauvignon and Ugni blanc. The resulting spectra showed similar ^13^C spectral fingerprints composed of cellulose, hemicelluloses, and lignins (Fig. 2a). The peaks allow the identification of most chemical groups of these three polymers based on previously reported data^32,33^. By comparing the total peak intensity of the control samples with samples inoculated with *F. mediterranea*, we determined the total loss of wood (Fig. 2b), which was estimated to be ~ 10% for Cabernet Sauvignon and ~ 7% for Ugni blanc. Similar experiments using Merlot showed no detectable losses (data not shown). Thus, the NMR results were in line with the C/N ratio analysis, with *F. mediterranea* having a significant negative impact on Cabernet Sauvignon and Ugni blanc sawdust, whereas no noticeable effect was detected on Merlot sawdust.

### Degradation of *Ugni* blanc sawdust by F. mediterranea, *Paenibacillus* sp. (S231-2), and P. amylolyticus (S293) (applied individually or in combination)

As the wood degradation and the wood loss rates between 13 and 20 days of incubation with *F. mediterranea* was greater on Ugni blanc sawdust than with other cultivars, Ugni blanc was chosen to study the effects of the two selected bacterial strains on wood degradation.

#### *F. mediterranea*’s mycelial growth on grapevine wood sawdust

**Figure 3:**
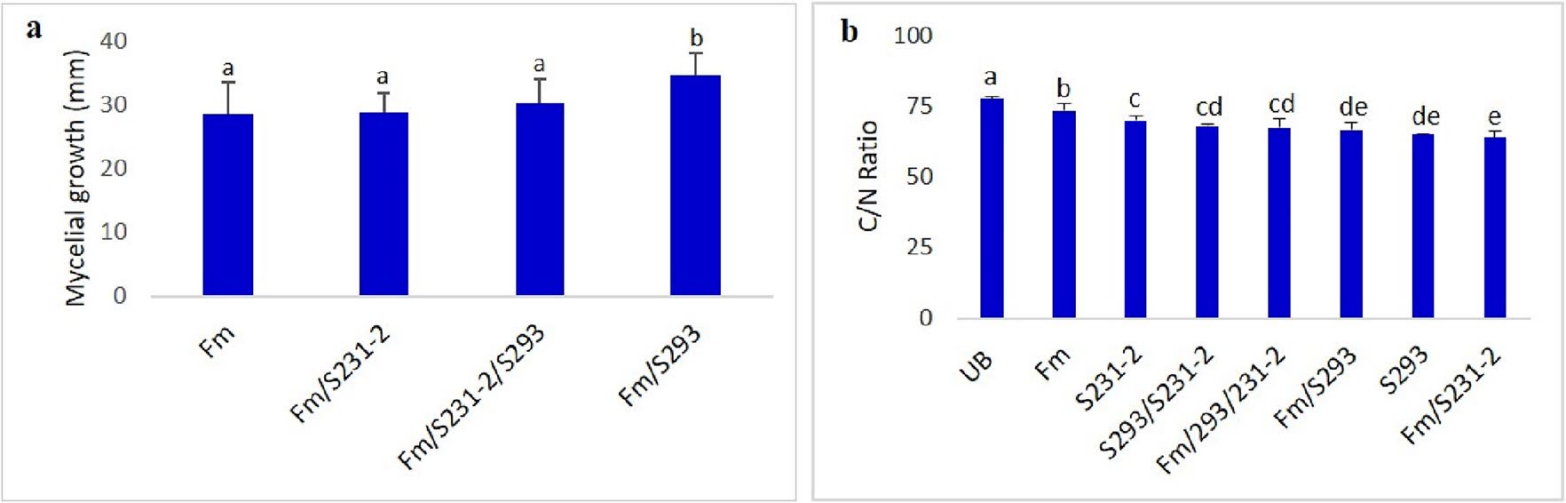
(**a**) Measurement of fungal mycelial growth on the sawdust of the different modalities inoculated with *F. mediterranea* (inoculated or not with bacterial strains). (**b**) C/N ratio at the end of the experimentation. UB: Ugni blanc, Fm: *F. mediterranea*, S231-2: *Paenibacillus* sp., S293: *P. amylolyticus*.

Mycelial growth of *F. mediterranea* was measured in all treatments inoculated with *F. mediterranea.* The results presented in Fig. 3a showed that *F. mediterranea*’s mycelial growth was more important in the presence of S231-2 and/or S293. Moreover, the co-inoculation of S293 with *F. mediterranea* strongly improved the mycelial growth of the pathogen.

### Degradation of Ugni blanc sawdust inoculated with F. mediterranea, Paenibacillus sp. (S231-2), and P. amylolyticus (S293) (inoculated individually or in combination)

As presented in Figs. 3b, S2 degradation in all treatments (inoculated with *F. mediterranea*) was significantly higher than that in the control (not inoculated). Interestingly, the bacterial strains inoculated alone (individually or in combination) significantly degraded grapevine wood powder, indicating that the bacteria could degrade grapevine wood into small fine particles. Based on the C/N ratio, the bacterial strains applied individually coupled with *F. mediterranea* to grapevine wood sawdust resulted in greater degradation than those inoculated with both bacterial strains coupled with *F. mediterranea* (Fig. 3b). The highest wood degradation was observed in wood co-inoculated with *Paenibacillus* sp*.* (S231-2) and *F. mediterranea* (Fig. 3b). However, the results observed above showed that mycelial development of the pathogen in the presence of strain S231-2 was less important compared to other modalities inoculated with *P. amylolyticus* S293 and *F. mediterranea* or with both bacterial strains and *F. mediterraena*.

**Figure 4:**
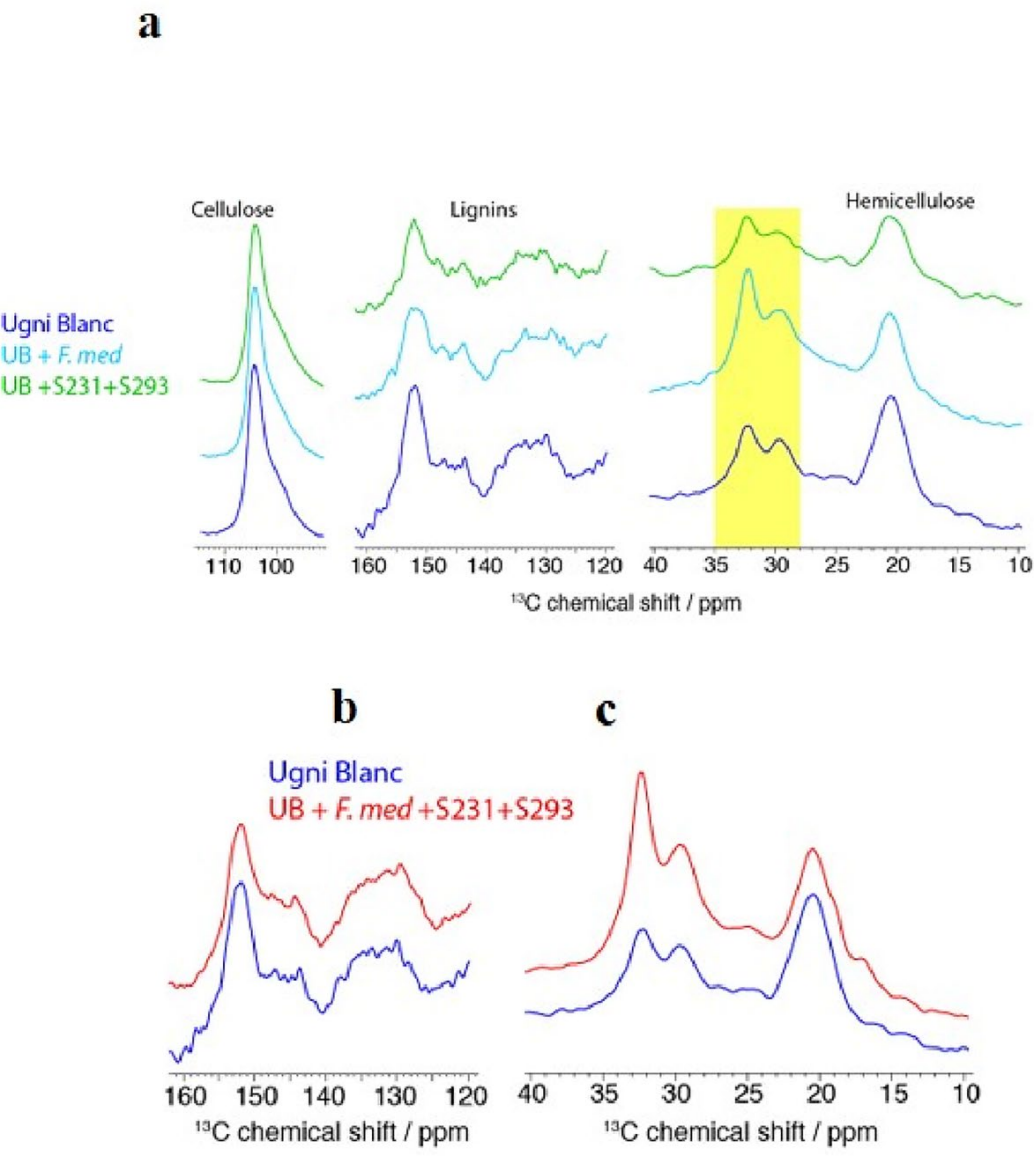
**(a**) Solid-state NMR wood decay characterization of Ugni blanc (UB) after inoculation of *F. mediterranea*, and *Paenibacillus* sp*.* (S231-2) and *P. amylolyticus* (S293). ^13^C CP spectra of UB (in blue) sawdust, inoculated with *F. mediterranea* (in cyan), and with a combination of S231-2 and S293 (in green). Spectral regions of cellulose, lignins and hemicelluloses are shown. The yellow rectangle corresponds to the chemical shift area of the degradation products. (**b**,**c**) Solid-state NMR wood decay characterization of Ugni blanc (UB) after inoculation of *F. mediterranea*, with *Paenibacillus* sp*.* (S231-2) and *P. amylolyticus* (S293). ^13^C CP spectra of Ugni blanc (UB) (in blue) sawdust and inoculated (in red). Spectral regions of lignins and hemicelluloses are shown.

To analyze the effect of wood degradation more precisely, we complemented the quantitative C/N ratio analysis with an investigation using MAS NMR on the effect of *F. mediterranea* and bacterial strains S231-2 and S293, and their combination on Ugni blanc sawdust. Because the chemical shifts of cellulose, hemicellulose, and lignin can be identified in ^13^C CP experiments, the evolution of each biopolymer can be monitored under different inoculation conditions. Figure 4a shows various spectral regions of ^13^C CP experiments recorded on a control sample of Ugni blanc sawdust compared to sawdust inoculated with *F. mediterranea* and a combination of *Paenibacillus* sp*.* (S231-2) and *P. amylolyticus* (S293). The cellulose contribution was clearly observed at ~ 105 ppm, which corresponded to the chemical shift of the C^13^ carbon (Fig. 4a). We observed comparable cellulose contributions among the three samples, as measured by the peak intensity, between the three samples. This indicates that wood degradation by *F. mediterranea* and the combination of S231-2 and 293 weakly affected cellulose. In contrast, two other major wood biopolymers, lignin and hemicellulose, showed noticeable changes between the three conditions. The contribution of lignin was observed in the spectral region of ~ 125–155 ppm, corresponding to ^13^C resonances of the aromatic groups of the guaiacyl, syringyl, and hydroxyphenyl units. A decrease in lignin contribution was detected, as indicated by a less intense signal (Fig. 4a). We estimated a decrease of ~ 35–40% in the lignin contribution (compared to cellulose) between the control sample and the sample inoculated with *F. mediterranea*. This observation is in line with the early NMR studies by Davis et al^34^., who reported preferential lignin degradation by white-rot fungi^34^. Interestingly, a comparable decrease (~ 35–40%) was observed between the control and samples inoculated with strains S231-2 and S293, suggesting that these bacteria also have the ability to degrade lignin. The degradation of hemicellulose was investigated by monitoring the signal at ~ 20 ppm, which corresponded to the methyl carbons of the acetyl groups. We detected a decrease in signal intensity of ~ 15–20% (compared to cellulose) between the control sample and the sample inoculated with *F. mediterranea* (Fig. 4a). The decrease was more significant for the samples inoculated with strains S231-2 and S293, with an estimated loss of intensity of ~ 20–25% (as compared to cellulose). In addition, we detected an increase in the signal in the spectral region of ~ 28–35 ppm (highlighted in yellow in Fig. 4a). Although precise chemical identification was not possible based on previous studies, we speculated that this signal contribution was related to the presence of CH_2_ groups that were not bound to oxygen^34,35^. This was likely due to the degradation products of hemicelluloses or products obtained after the reductive depolymerization of lignins. Overall, our results indicate a noticeable degradation of lignins and hemicelluloses after inoculation, which was more pronounced for lignins.

The synergistic effect of fungi and bacteria was investigated using MAS NMR experiments on Ugni blanc sawdust inoculated with a combination of *F. mediterranea* and bacterial strains S231-2 and S293. Following the same approach based on NMR signal comparison between the inoculated and control samples, we studied the degradation of the biopolymers. At the lignin level (Fig. 4b), a small decrease of ~ 5% was detected, suggesting that the combination of *F. mediterranea* with strains S231-2 and 293 had a different degradation effect on sawdust than inoculation with the fungus or bacteria alone. The same observation was made for hemicelluloses with a signal decrease of ~ 10% (Fig. 4b,c), while the signal pattern of cellulose was similar in the control and inoculated samples (data not shown). The most important difference was observed in the spectral region of ~ 28–35 ppm, for which a drastic increase of ~ 50–60% (as compared to cellulose) was observed (Fig. 4b,c). Overall, the degradation effect of bacteria and fungi did not result from the simple addition of their degradation on each biopolymer, but this quantity of degradation products and depolymerized lignins greatly increased when fungal and bacterial communities were used in synergy.

### Genome analysis of S231-2 and S293

#### Genomic features, taxonomic affiliation and COG categories

**Figure 5:**
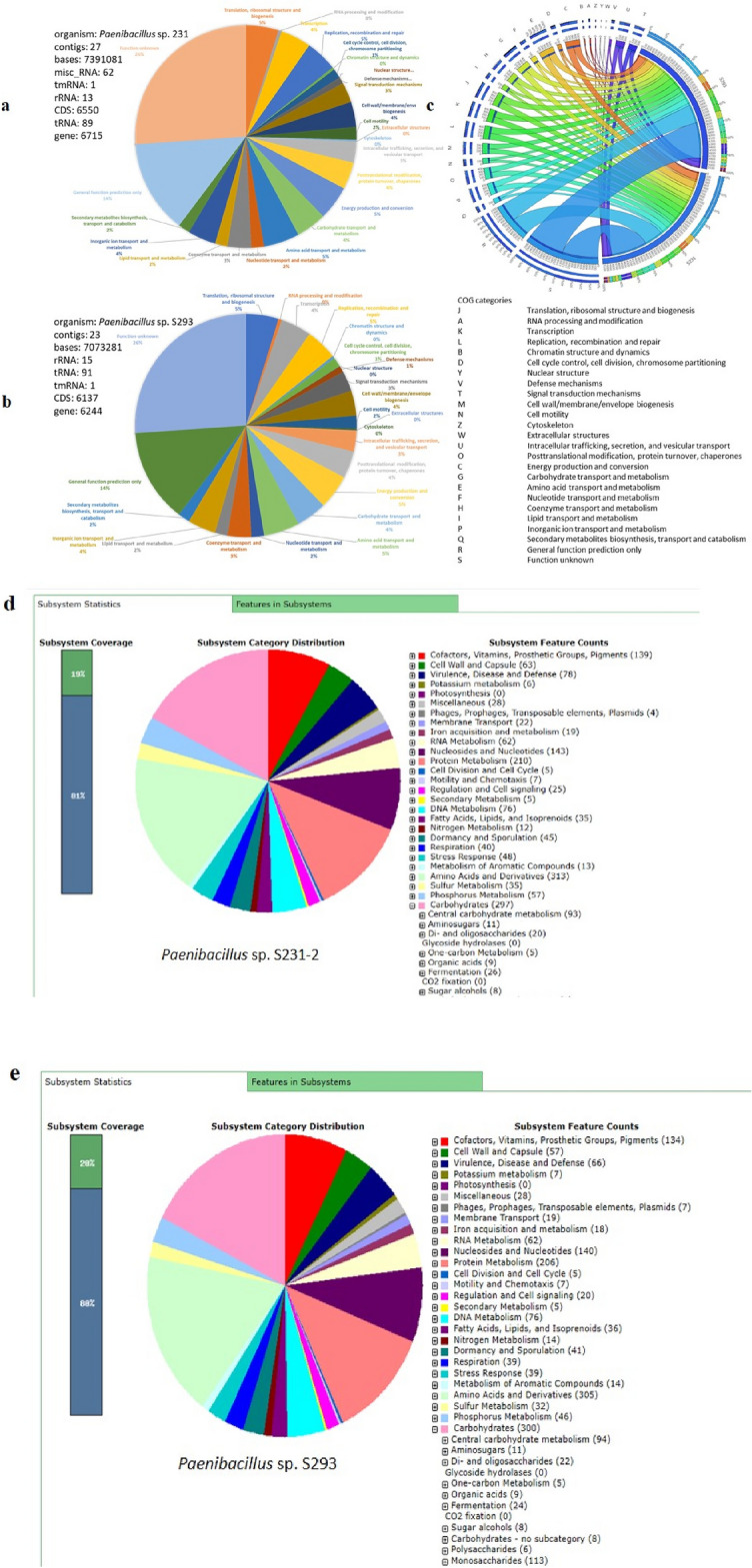
Genome annotation of S231-2 and S293 showing COG categories for each strain, a circus simulation to compare COG categories between strains as well as RAST annotation (enabling to see each subcategories including the numbers of genes related to xylose/xyloside degradation).

The genomes of the strains S231-2 and S293 were sequenced, assembled, and annotated. Each genome had a total size of 7.39 Mb and 7.07 for S231-2 and S293, respectively, as well as an average G + C content of 45.7% and 45.8% and 6,715 and 6,244 coding genes, respectively (Fig. 5). The assessment of genome quality based on 468 markers showed a completeness of 99.85% for both strains with no contamination. The taxonomic placement based on Average Nucleotide Identity (ANI) provided a match with *P. amylolyticus* with a radius of 95.25%. The number of genes in each COG category did not change among the strains (Fig. 5a,b), as revealed by the Circos simulation (Fig. 5c). However, 28 genes associated with subsystems (Fig. 5d,e) in strain S231-2 were not predicted in strain S293, and 18 genes detected in S293 were not found in the genome of S231-2 (Tables S3, S4).

#### Genes predicted to be involved in lignin, cellulose and hemicellulose degradation

In addition to other clusters of ortholog groups, several genes involved in carbohydrate transport and metabolism were predicted using RAST or eggnog annotation. Genes possibly involved in lignin degradation (related to lignin-degrading auxiliary enzymes and lignin-modifying enzymes) were detected in the S231-2 and S293 genomes (Tables 1, S1, S2, and S5-S6-S4). During the initial stages of lignin degradation, extracellular enzymes are responsible for lignin depolymerization. Lignin-modifying enzymes (LME) and lignin-degrading auxiliary (LDA) enzymes are the two main groups of lignin-degrading enzymes^36^. Multiple lignin modification- and depolymerization-related genes were screened in the two genomes, including genes encoding LME, that is, small laccase-like multicopper oxidase genes, other laccases, dye-decolorizing peroxidase genes, manganese peroxidase-encoding genes, versatile peroxidases, and genes encoding LDA, such as glyoxal oxidases, aryl alcohol oxidases, glucose dehydrogenases, pyarnose 2- oxidases, cellobiose dehydrogenases, and cytochrome P450. Using eggnog annotation and for strain 293: 2 multicopper oxidases, 15 glyoxal oxidases, 1 aryl alcohol oxidase, 1 glucose dehydrogenase, and 7 cytochrome P450 related genes were detected. In strain S231-2, 3 multicopper oxidases, 14 glyoxal oxidases, 1 aryl alcohol oxidase, 2 glucose dehydrogenases, and 8 cytochrome P450 related genes were detected (Tables 1 and S5-S6).

**Table 1:** Number of genes of strains S231-2 and S298 putatively involved in lignin degradation, after analyzis using eggnog annotation and functionally annotated genes.

	Number of genes		
	S231-2	S293	S231-2/S293^a^
Lignin-modifying enzymes			
Small laccase-like multicopper oxidases	3	2	
1	S231-2_06045	–	–
2	S231-2_02561	S293_02831	97%
3	S231-2_03245	S293_02343	97%
Other laccases	–	–	
Dye-decolorizing peroxidases	–	–	
Manganese peroxidases	–	–	
Versatile peroxidases	–	–	
Lignin-degrading auxiliary activities			
Glyoxal oxidases	14	15	
Aryl alcohol oxidases	1	1	
Heme-thiolate haloperoxidases	–	–	
Glucose dehydrogenases	2	1	
Pyranose 2-oxidases	–	–	
Cellobiose dehydrogenases	–	–	
Cytochrome P450	8	7	

^a^Identities on protein level of *Paenibacillus* sp. S231-2 compared to S293.

**Figure 6:**
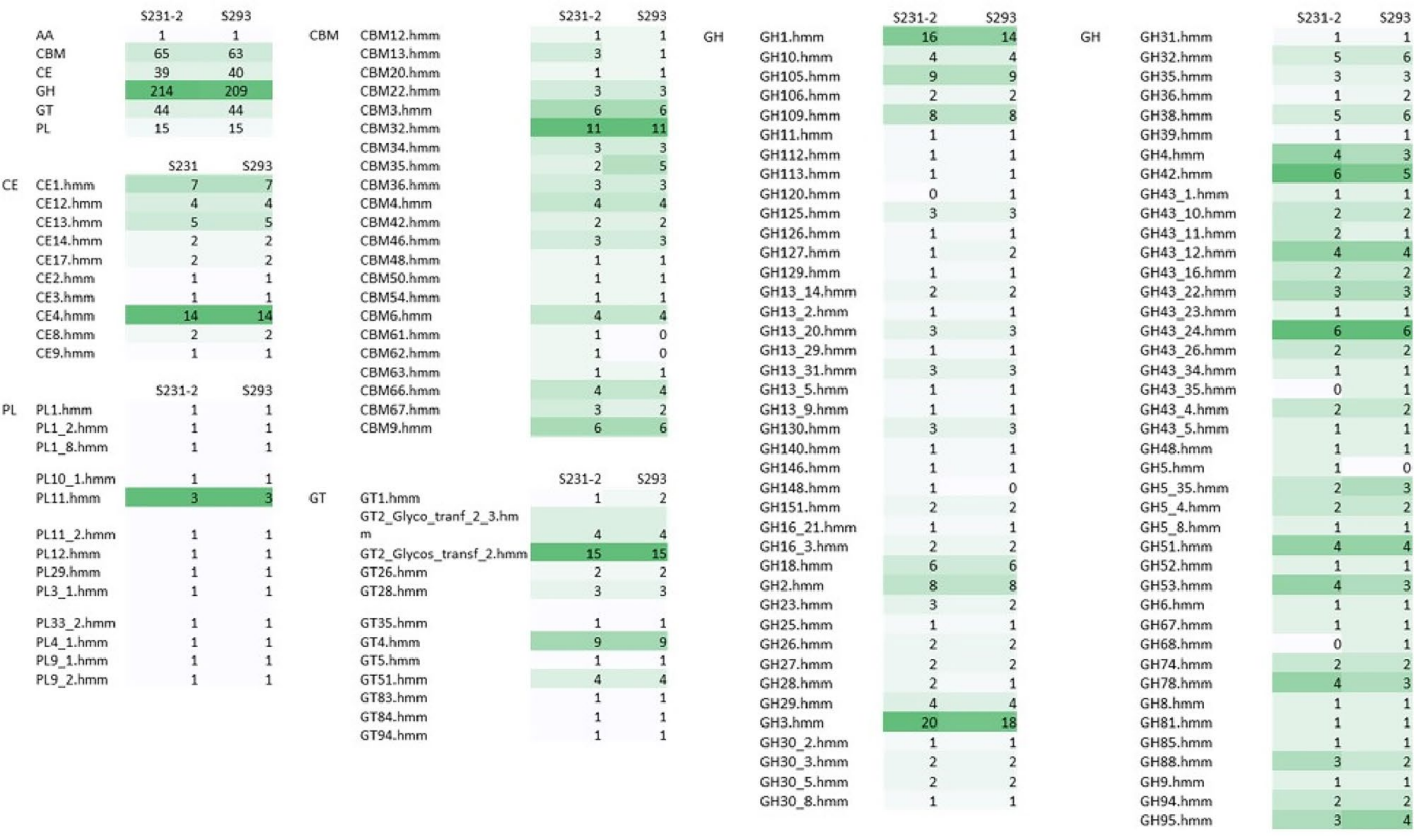
Carbohydrate-active enzymes (CAZymes) related genes of strain S231-2 and S293 assessed with dbCAN2. CBM: carbohydrate-binding modules, CE: Carbohydrate esterases, GT: Glycosyltransferases, PL: Polysaccharide lyases, AA. Auxiliary activities.

Furthermore, we analyzed all gene clusters related to carbohydrate-active enzymes (CAZy) to gain a better understanding of the functions of carbohydrate-related genes encoding auxiliary activities, carbohydrate-binding modules, carbohydrate esterases, glycoside hydrolases, glycosyltransferases, and polysaccharide lyases (Fig. 6). In the CAZyme database, lignin-degrading enzymes are subdivided into the AA class, which are redox enzymes that act in conjunction with CAZymes. Lignin-oxidizing enzymes (LO) are in the AA1, AA2, and AA3 classes, and lignin-degrading auxiliary enzymes (LD) in the AA4, AA5, AA6, and AA8 classes. Genome analysis using the CAZyme database predicted that strains S231-2 and S293 had only one AA-related gene (AA7) that was not associated with lignin degradation. Using CAZyme annotation, we could not detect any genes related to lignin degradation. However, it is known that bacterial genes involved in lignin degradation are not included in the CAZyme database, except if their products carry a carbohydrate-binding module (CBM)^37^.

Genome analyses of strains S231-2 and S293 predicted several genes in each strain related to glycosyl hydrolase (GH) families, as well as subfamilies, such as GH1, GH105, GH18, GH2, GH3, and GH32 (with all having several different activities), GH38 (mannosidase, EC 3.2.1.-), GH42 (β-galactosidase, EC 3.2.1.23 or α-L-arabinopyranosidase, EC 3.2.1.-, GH43_24 (exo-β-1,3-galactanase, and EC 3.2.1.145) containing at least five sequences for each strain (Fig. 6). All of these glycosyl hydrolases are involved in the degradation of hemicellulose (with glucosidase, xylosidase, mannosidase, galactase, rhamnase, and arabinase) or in cellulose degradation (such as GH1 and GH3). Other genes related to the CBM, PL (Polysaccharide lyases), CE (Carbohydrate esterases) and GT (Glycosyltransferases) were detected in each genome (Fig. 6). Six genes involved in xylose (xyloside) degradation were further predicted in each genome, with *Xyl*A coding for a xylose isomerase that catalyzes the conversion of d-xylose to d-xylulose, *Xyl*B coding for a xylulose kinase that catalyzes the phosphorylation of d-xylulose to d-xylulose 5-phosphate, *XynT* coding for a xyloside transporter, and xylosidase for both strains (Tables S1-S2).

### Genes predicted to be involved in plant–microbe and fungi-*bacteria* interaction

Using RAST and eggnog annotation, analysis of the two bacterial genomes enabled the detection of protein-encoding genes that were predicted to be involved in plant–microbe and microbe-microbe interactions, including motility and chemotaxis, biofilm formation, sugar and nutrient metabolism, siderophore and iron transport, auxin synthesis, nitrogen metabolism, as well as genes related to osmotic stress, oxidative stress, detoxification, and several other genes (Tables S1-S4). Interestingly, the strains S231-2 and S293 possessed several genes that can contribute to arsenic resistance. Several gene clusters involved in the metabolism of vitamins, such as biotin (B7), thiamine (B1), pyridoxine (B6), cobalamine (B12), and phylloquinone (K2), were also detected during the analyses of the genomes of strains S231-2 and S293, but with a higher number of predicted genes for S293 (Tables S1-S2 and S5-S6). According to previous studies, some of these genes, such as those involved in vitamin B production, may also be involved in fungal growth stimulation^38,39^.

### Biosynthetic gene clusters involved in secondary metabolite production

A total of 17 and 16 gene clusters likely involved in secondary metabolite production were identified in strains S231-2 and S293, respectively, using AntiSmash 7.0^40^. The core biosynthetic genes in the 16 common clusters were 94% or more identical at the protein level, indicating homologous biosynthesis clusters in the strains S231-2 and S293 (Table 2). Of these, one non-ribosomal peptide synthetase (NRPS) cluster encodes a polymyxin type, as indicated by > 90% identity at the protein level of the biosynthesis genes compared to the verified polymyxin producer *P. alvei*^41^. One NRPS polyketide synthase (PKS)- hybrid cluster has 50–60% identity at the protein level to paenilarvin of *P. larvae*^42^ in three genes, but the fourth is missing in strains S231-2 and S293 (Table 2). Therefore, a metabolite with similarities to paenilarvin is possible in S231-2 and S293, but the exact structure is certainly different. The complete gene cluster encoding for the siderophore bacillopaline from *P. mucilaginosus*^43^ is more than 60% identical, on protein level, in both strains S231-2 and S293. The production of bacillopaline, a closely related siderophore, is likely to occur in both strains.

**Table 2:** Predicted secondary metabolites in *Paenibacillus* sp. S231-2 and S293.

Compound	Query proteins^a^	S231-2			S293			S231-2/S293^b^
		Protein ID	Coverage	Identity	Protein ID	Coverage	Identity	
(1) NRP:Polymyxin/Colistin (*P.alvei*)	LR_PmxE	S231-2_05531	100%	92%	S293_05341	100%	92%	99%
	LR_PmxD	S231-2_05530	100%	97%	S293_05342	100%	97%	99%
	LR_PmxC	S231-2_05529	100%	95%	S293_05343	100%	94%	97%
	LR_PmxA	S231-2_05527	100%	91%	S293_05345	100%	91%	98%
	LR_PmxB	S231-2_05528	100%	91%	S293_05344	100%	91%	98%
(2) NRP: Paenillarvin (*P. larvae* ssp. *larvae*)	AHD05677_NRPS	S231-2_03165	90.90%	49%	S293_02262	90.90%	49%	98%
	AHD05678_NRPS	S231-2_03165	61.10%	51%	S293_02262	61.10%	51%	98%
	AHD05679_NRPSPKS	S231-2_03166	100%	55%	S293_02263	100%	55%	98%
	AHD05680_malonyl-transacylase	S231-2_03167	97.70%	60%	S293_02264	97.70%	60%	99%
(3) Siderophore: Bacillopaline (*P. muci-laginosus*)	KNP414_01893_HP	S231-2_04961	99.30%	62%	S293_04966	99.30%	62%	100%
	KNP414_01894_HP	S231-2_04962	93.80%	60%	S293_04965	93.80%	60%	100%
	KNP414_01895_HP	S231-2_04963	96.60%	66%	S293_04964	96.60%	67%	98%
	KNP414_01896_ABC	S231-2_04964	100%	72%	S293_04963	98.90%	73%	97%
	KNP414_01897_ABC	S231-2_04965	99.70%	73%	S293_04962	99.70%	72%	99%
	KNP414_01898_ABC	S231-2_04966	93.90%	72%	S293_04961	93.90%	72%	99%
	KNP414_01899_ABC	S231-2_04967	97.70%	70%	S293_04960	97.70%	70%	99%
	KNP414_01900_ABC	S231-2_04968	100%	64%	S293_04959	100%	64%	97%
	KNP414_01901_HP	S231-2_04969	97.80%	69%	S293_04958	100%	67%	99%
Other AntiSmash 7.0 predictions:		Region in S231-2^c^			Region in S293			
(4) NRPS^d^		S231-2_01215			S293_00659			99%
(5) NRPS		S231-2_04511			S293_03180			99%
(6) NRPS-PKS^e^ hybrid		S231-2_02719, S231-2_02720			S293_02986, S293_02987			98–99%
(7) NRPS-PKS hybrid		S231-2_04595, S231-2_04593, S231-2_04592, S231-2_04589			S293_03268, S293_03266, S293_03265, S293_03262			99%
		S231-2_06456, S231-2_06457, S231-2_06458, S231-2_06459			S293_06028, S293_06029, S293_06030, S293_06031			
(8) NRPS-PKS hybrid		S231-2_00526			S293_01351			99–100%
		S231-2_00790			S293_01088			
(9) Type 3 PKS		S231-2_01510			S293_00361			99%
(10) Type 3 PKS		S231-2_04083			S293_04410			100%
(11) RiPP^f^: Lassopeptide		S231-2_05316			S293_04789			100%
(12) RiPP: Proteusin		S231-2_05427			S293_04680			100%
(13) RiPP: lanthipeptide		S231-2_02093			n.d			99%
(14) RiPP: lanthipeptide		S231-2_04289; S231_04292; S231-2_04293			S293_04215, S293_04212, S293_04211			98%
(15) Cyclic-lactone-autoinducer		S231-2_03531			S293_03993			–
(16) Siderophore: IucA/IucC-like								94–100%
(17) Terpene								98%

Capacity of the production of NRPs, polymyxin and a paenilarvin-like compound and the siderophore bacillopaline as well as predicted secondary metabolites by antiSMASH 7.0.

^a^Identities and coverage of complete cluster predicted by AntiSmash 7.0 are shown as identities in comparison to the known producers *P. alvei* LR, *P. larvae* ssp. *larvae* DSM 25,430 and *P. mucilaginosus* KNP414.

^b^Identities on protein level of *Paenibacillus* sp. S231-2 compared to S293.

^c^Core synthesis proteins of the potential secondary metabolite clusters.

^d^NRPS: non-ribosomal peptide synthetase.

^e^PKS: polyketide synthase.

^f^RiPP: ribosomally synthesized and post-translationally modified peptides.

The identities of the potentially produced metabolites in the remaining secondary metabolite clusters are unknown. These additional metabolites include two NRPS, three PKS-NRPS hybrids, two type 1 PKS, one siderophore, one terpenoid (potentially involved in carotenoid production), and four ribosomally synthesized and post-translationally modified peptides (RiPPs). Strain S231-2 contains additionally a cyclic lactone autoinducer peptide (Table 2). Raw sequence data for the bacterial genomes of strains S231-2 and S293 have been deposited in the National Center for Biotechnology Information (NCBI). They can be accessed under the name BioProject ID PRJNA1015190.

## Discussion

Fungi have historically been considered as the dominant pathogens involved in wood decay because of their ability to degrade lignocellulosic biopolymers^44,45^. The role of Ascomycota in grapevine wood disease has been extensively studied, but there is a lack of information regarding Basidiomycota members, especially regarding their relationship with other microorganisms, such as bacteria. The mode of action of the basidiomycete, *F. mediterranea*, which is responsible for the development of white-rot, a key necrotic factor involved in Esca disease, has been recently reviewed^10^. The non-enzymatic process for destroying wood components is still being investigated^10^, and at the same time the synergistic relationship of bacteria with this fungus has also been initiated^15^.

In this study, we examined the susceptibility of three grapevine cultivars to *F. mediterranea*. Our results obtained with a microcosm system are in agreement with data in the literature reporting that the cultivar Ugni blanc is more susceptible to Esca disease than Cabernet Sauvignon and Merlot^46,47^. The NMR data suggest that *F. mediterranea* degrades lignins and hemicelluloses more intensely than cellulose. Basidiomycetes have sophisticated processes to selectively degrade lignin species^48,49^ and here, we report a notable loss of lignin after inoculation with *F. mediterranea*. Overall, we can hypothesize that in the vineyards, the difference in Esca-susceptibility between the tested cultivars could be explained, at least partly, by the different levels of wood degradation caused by *F. mediterranea* activity.

The roles of bacteria and their association with *F. mediterranea* in the degradation of grapevine wood were investigated. Because of the presence of numerous bacteria in all grapevine wood tissues, that are, healthy and necrotic^50^, their role in Esca needs to be investigated to better understand this Esca pathosystem. Because we showed that Ugni blanc was the most susceptible cultivar, its sawdust was used as a model to study the role of cellulolytic and xylanolytic bacterial strains in wood degradation. To the best of our knowledge, we showed for the first time that two bacterial strains of the genus *Paenibacillus* (S231-2 and S293) could increase the degradation of Ugni blanc wood colonized by *F. mediterranea.* Both strains were isolated from cordon wood tissues of Sauvignon blanc cultivar^15^. The highest wood degradation was obtained with the association of *F. mediterranea* with the bacterial strain S231-2 (*Paenibacillus* sp.). A similar increase in Cabernet Sauvignon sawdust degradation was obtained by the co-inoculation of another strain of *Paenibacillus* (S150) with *F. mediterranea*. These results confirm those obtained with other bacteria that enhance the degradation of wood from other plants by wood-rotting basidiomycetes, such as *Trametes versicolor* and *Phanerochaete chrysosporium*^21–24^. For example, the co-culture of the fungus *T. versicolor* with *Cupriavidus* sp. TN6W-26 and *Enterobacter* sp. TN6W-26 enhanced the lignin degradation activity of this fungus^21^.

NMR data indicated a slight preference for hemicellulose degradation when comparing bacterial and fungal inoculations. We speculate that because of the presence of shorter chains and exposed sugar moieties, hemicelluloses are relatively easy to degrade, and our results suggest that bacterial strains S231-2 and 293 are more prone to degrade them.

Hoppe et al^51^ reported that the degradation of wood polymer structures increased the wood moisture content and the access of microorganisms to the wood. As bacteria have been reported to benefit from high wood moisture, thereby contributing to its decomposition^52^, the ability of bacterial strains S231-2 and/or S293 to degrade Ugni blanc grapevine wood on their own could also be explained by the increasing water availability in the degraded wood. Moreover, numerous genes which encode for enzymes involved in xylose (xyloside), cellulose, hemicellulose, and lignin degradation and modification were detected in the genomes of strains S231-2 and S293.

Although the greatest degradation was observed when S231-2 (*Paenibacillus* sp.) was co-inoculated with *F. mediterranea* on Ugni blanc sawdust, this was not associated with an increased growth of *F. mediterranea* mycelia, unlike the increased mycelial growth observed in the presence of strain S293. We hypothesized that the bacterial strains interacted differently with *F. mediterranea* during wood degradation processes; S231-2 may predispose sawdust to fungal attacks, and S293 (*P. amylolyticus*) may directly improve fungal growth. Among the possible modes-of-action of bacteria, it has been reported that bacteria can stimulate the fungal wood degradation by producing fungal growth-promoting substances, especially vitamins, and/or by stimulating certain enzymatic activities through the production of degradation effectors^53,54^. Our results are consistent with this, as we found genes which encode products related to vitamin metabolism in the genome of each strain.

In addition, the complete genome provided further evidence for bacterial strains abilities to degrade wood components. In the two genome sequences, numerous genes encode enzymes potentially involved in the degradation of cellulose, hemicellulose and lignin. For instance, the presence of genes related to lignin-degrading auxiliary enzymes and lignin-modifying enzymes, which are possibly involved in lignin degradation, are correlated with the results obtained in the genomes of other lignin degrading bacteria, such as some strains of *Bacillus subtilis*, *Erwinia billingiae* and *Klebsiella variicola*^55–57^.

Both strains encode also secondary metabolites such as siderophores and NRPS polymyxin. These compounds specifically act against a group of microorganisms, especially gram-negative bacteria^58^, and the secondary metabolite and siderophore potential may be involved in shaping the community in Esca-infected tissues.

In wood microcosms, white-rot fungi have been reported to induce significant changes in the bacterial community composition^23,59^, and promote the growth of cellulolytic and xylanolytic bacterial strains with less inhibitory effects against these fungi^22^. In line with these observations, our NMR data suggest that the synergistic effect of *F. mediterranea* with strains S231-2 and S293 is not just a simple addition of their degradation ability, but that the increase in degradation products is higher when bacteria and fungi are inoculated together on Ugni blanc. In addition, NMR data showed that the signal intensities differed between sawdust inoculated with bacteria or *F. mediterranea*. A higher contribution was observed for the sample inoculated with this fungus, suggesting different degradation mechanisms for the two samples. This is consistent with known degradation mechanisms that differ between fungi and bacteria, due to the ability of the former to produce more wood-degrading enzymes^36,49^. Overall, our results indicate a notable degradation of lignins and hemicelluloses after inoculation, which was more pronounced for lignins.

The increase in Ugni blanc sawdust degradation compared to Cabernet Sauvignon and Merlot sawdust was not correlated with sawdust/wood colonization by *F. mediterranea.* Slower colonization associated with more efficient and faster wood degradation could explain this result.

All the results obtained with the three bacterial strains, S150^15^, S231-2, and S293, confirmed our hypothesis that some bacteria colonizing grapevines enhance the ability of fungi to degrade wood structures. It was also shown that these three bacterial strains, which directly degrade wood components, share a common feature: they belong to the *Paenibacillus* genus. Various species of *Paenibacillus* are able to produce glucanases, cellulases, chitinases, xylanases, and proteases that are implicated in the destruction of eukaryotic cell walls^60–62^. Recently, Tahir et al^63^ identified three strains of *Paenibacillus* sp. that degrade lignin, and they showed that *Paenibacillus* enzymes degrade and/or modify this wood biopolymer.

The results obtained in this study contribute to the current evidence that bacteria interact with fungi during wood decay^16,64^. For example, laccase-like multicopper oxidases have recently been associated with lignin degradation in several Gram-positive bacteria^65^, and similar genes, such as those related to small laccase-like multicopper oxidases, have been identified in the *Paenibacillus* strains S231-2 and S293, supporting their potential function in lignin degradation or modification, although the specific function of these proteins requires further characterization.

In conclusion, our results show that two *Paenibacillus* strains are involved alone in the degradation of grapevine wood, but their association with the fungus, *F. mediterranea*, increased this wood degradation. *F. mediterranea* and bacterial degradation pathways of different grapevine cultivars will provide answers to understand the role of microbial communities in Esca and GTDs pathosystems, and to propose more efficient control management. To determine the frequency of these microbial interactions in the wood of mature grapevines would also be relevant in the future.

## Methods

### Microorganisms and culture conditions

#### Selected bacteria and bacterial inoculum

The two bacterial strains used were selected from a previous experiment on wood microcosms (i.e., the medium was made of sawdust grapevine), as they displayed strong cellulase and xylanase activities and did not inhibit the growth of *F. mediterranea* mycelia^15^.

For inoculations, the bacterial suspensions of strain S231-2 (*Paenibacillus* sp.) and S293 (*P. amylolyticus* were prepared as follows: Liquid cultures were obtained by inoculating Erlenmeyer flasks containing TSB with bacterial colonies pre-grown on TSA and then by incubating them at 28 °C for 24 h using an orbital shaker at 150 rpm. Liquid cultures were then centrifuged twice at 5000 rpm for 10 min, and the pellets were resuspended in sterile water to obtain liquid suspensions for use in the microcosm experiments. The bacterial concentration of each suspension obtained was estimated at 2 × 10^8^ CFU mL^−1^.

#### Fomitiporia mediterranea strain and culture conditions

*F. mediterranea* strain (PHCO36) used in this study was obtained from the INRAE-UMR 1065 SAVE collection (Bordeaux, France). The fungus was stored at 4 °C on Malt Agar (MA) medium. The cells were subcultured on MA and incubated at 28 °C for 7 d before use in the microcosm experiments.

### Plant material

Grapevine cuttings of Cabernet Sauvignon, Merlot, and Ugni blanc cultivars, originating from INRAE experimental vineyards, were used for the microcosm experiments. Depending on the experiment, the wood of each cultivar was crushed and sieved (1-mm mesh to obtain sawdust) and then autoclaved (20 min, 120 °C) twice, for 2 days in between. Sawdust was plated on TSA and MA media to determine the initial sterility. After incubation at 28 °C for 7 days, no microbial growth was detected.

### Microcosm experimentations

#### Susceptibility of three cultivars of grapevine to *F. mediterranea*

##### Experimental design

Experiments were carried out with *F. mediterranea* in microcosms (90 mm Petri plates sealed with adhesive tape) containing 2 g of sawdust wood from each cultivar. A mycelial disk (5 mm in diameter) taken from the margin of a 7-day-old fungal colony was placed on sawdust and inoculated with 5 mL of sterile distilled water. Control plates were inoculated with 5 mL of sterile distilled water only. Each plate was sealed with transparent adhesive tape and incubated for 13 or 20 d at 28 °C in the dark. Two treatments, each applied to 35 microcosms, were tested as follows: (i) controls containing sawdust from each cultivar with sterile water, and (ii) experimental group containing sawdust from each cultivar with *F. mediterranea* and sterile water.

##### Fungal growth measurement

To evaluate any possible variations in the growth of *F. mediterranea* on the wood of the three cultivars, fungal growth was assessed by measuring mycelial growth in all replicates of each treatment (35 microcosms by cultivar) on days six and nine post inoculation.

##### Estimation of the wood degradation

After 13 days of incubation, the degradation of sawdust in four micocosms was assessed by measuring the C and N content in the wood by the Dumas method using a VarioMax cube elemental analyzer at the USRAVE precincts (USRAVE, INRAE Aquitaine, France). The C/N ratio provides information on changes in the chemical composition of organic matter. This ratio is an important variable correlated with organic matter mass loss, especially during the decomposition process^22,23,66–69^. The size and color of the sawdust were observed and compared with those of the corresponding control.

### Effect of P. amylolyticus (S293), Paenibacillus sp. (S231-2), and F. mediterranea co-culture on Ugni blanc wood degradation

#### Experimental design

Ugni blanc sawdust was used to study the effects of two bacterial strains, S231-2 (*Paenibacillus* sp.) and S293 (*P. amylolyticus*), on wood degradation, with or without *F. mediterranea*. The inoculation of *F. mediterranea* was done as previously described. For bacterial inoculation, 5 mL of the bacterial cell suspension was added to the microcosms inoculated with only one bacterial strain. In the microcosms inoculated with both bacterial strains, only 2.5 mL of each bacterial cell suspension was added. Each plate was sealed with transparent adhesive tape and incubated for 13 d at 28 °C in the dark.

Eight treatments, each applied on 25 microcosms, were tested (i) control containing Ugni blanc sawdust with sterile water; or Ugni blanc sawdust containing: (ii) *F. mediterranea* with sterile water; (iii) *F. mediterranea* and *P. amylolyticus* (S293) or *Paenibacillus* sp. (S231-2); (iv) *F. mediterranea*, *P. amylolyticus* (S293), and *Paenibacillus* sp. (S231-2); (v) *P. amylolyticus* (S293) or *Paenibacillus* sp. (S231-2); (vi) *P. amylolyticus* (S293) and *Paenibacillus* sp. (S231-2).

### Effects of *Paenibacillus* sp. (S231-2) and/or P. amylolyticus (S293) on the growth of F. mediterranea

To evaluate the effect of bacterial inoculation on the mycelial growth of *F. mediterranea* on the wood in the eight treatments described above, fungal growth was assessed by measuring the mycelial growth of all repetitions (25 microcosms) on day six post inoculation.

### Estimation of the wood degradation by measurement of C and N concentrations

Wood degradation was estimated by measuring the C and N concentrations in the sawdust of six microcosms after treatment at 13 dpi (day post inoculation) using the Dumas method with a VarioMax cube elemental analyzer at the USRAVE precincts (USRAVE, INRAE Aquitaine, France).

### Statistical analyses

The experimental data obtained from microcosm experimentations, were compared using analysis of variance (ANOVA), followed by the Newman–Keuls test (*P* = 0.05). These analyses were carried out with the STATBOX software (Version 6.6, grimmer Logiciels, Paris, http://www.statbox.com).

#### Estimation of the wood degradation by solid-state (NMR) spectroscopy

MAS solid-state NMR spectroscopy experiments were performed on one microcosm a 7 T (300 MHz ^1^H Larmor frequency) Avance III spectrometer (Bruker Biospin) using a 4 mm dual CP-MAS DVT N-P/H probe. The MAS frequency was set to 11 kHz and the probe temperature was set to 6.85 °C. Proton decoupling (90 kHz, SPINAL-64) was applied during ^13^C direct acquisition. 1D ^13^C cross-polarization (CP) experiments (5120 scans) were performed using a contact time of 500 µs.

### Genome contents of the bacterial strains Paenibacillus sp. (S231-2) and P. amylolyticus (S293)

The bacterial genomic DNA of each strain was extracted following a phenol–chloroform-based protocol after growing the strain in liquid TSB medium for 3 days and harvesting it by centrifugation at 6000 rpm for 3 min (as reported by Haidar et al^15^ for strain S150). The bacterial pellets were resuspended in a lysis buffer (0.2 mg mL^−1^ Proteinase K, 50 mM Tris–Cl, 1% SDS, 5 mM EDTA at pH 8 and 0.5 M NaCl) and incubated overnight (at 65 °C; 400 rpm). DNA from each strain was extracted twice using phenol–chloroform–isoamyl alcohol at a ratio of 25:24:1 and collected by centrifugation at 6,000 rpm for 3 min, as described previously^38^. Genomic DNA was purified using Amicon Ultra 0.5 mL 30 K Centrifugal Filter Units (Millipore, Cork, Ireland) and resuspended in sterile distilled water. Whole-genome shotgun sequencing of each strain was performed using an Illumina NovaSeq 6000 mode S2 (GATC Biotech, Konstanz, Germany), producing approximately 6.2 million paired-end reads of 150 bp. The Illumina reads were screened for the presence of PhiX using Bowtie 2 (v2.3.4.3)^70^; adapters were trimmed, and quality filtering was performed using FASTP (v0.19.5)^71^. The sequence length distribution and quality were checked using FastQC^72^. Genome assembly was performed with SPAdes v3.13.0^72^, and low-abundant (< 2 ×) and short (< 500 bp) contigs were discarded. The contigs were checked for the presence of contaminants using BlobTools v.1.1.1. The quality of genome assemblies was determined using QualiMap v2.2^73^ and QUAST v5.0.0, and then genome completeness of the reconstructed genomes was evaluated using CheckM v1.2.2^74^. Gene annotation was carried out using Prokka v1.12^75^ and the NCBI Prokaryotic Genome Annotation Pipeline (PGAP). Plasmid presence was determined using Mash v2.1 against the PLSDB database^76^. Putative plasmid contigs were screened for the presence of genes encoding the replication initiator protein, *rep*A. For this purpose, a curated FASTA file with approximately 8,000 *rep*A genes was generated from the plasmid genome sequences in NCBI. These *repA* sequences were used to build a database against which selected contigs were aligned using BLAST + v.2.10.0. These *rep*A gene sequences were used to build a database against which the selected contigs were BLASTed. Functional annotation was performed using WebMGA for COG, the ClassicRAST (Rapid Annotation using Subsystem Technology) webserver (http://rast.nmpdr.org) ^77^ and the hierarchical orthology framework EggNOG 4.5^78^. The Carbohydrate-Active Enzyme (CAZy) families were ascertained using dbCAN2 based on the HMMER database. Proteins were annotated using the CAZy database^79^. Biosynthetic gene clusters and secondary metabolites were predicted using antiSMASH version 4.0.2^80^. To assign objective taxonomic classifications to the genome, the software toolkit GTDB-Tk v2.3.2 was used^81,82^ using the Genome Taxonomy Database release version 214 (https://gtdb.ecogenomic.org/).

### Supplementary Information

Supplementary Information 1.

Supplementary Information 2.

Supplementary Information 3.

## Data Availability

Raw sequence data for the bacterial genomes of strains S231-2 and S293 have been deposited in the National Center for Biotechnology Information (NCBI). They can be accessed under the name BioProject ID PRJNA1015190.
