# Supplementary material for: Two Paenibacillus spp. strains promote grapevine wood degradation by the fungus Fomitiporia mediterranea: from degradation experiments to genome analyses
Source: Sci Rep. 2024 Jul 9;14:15779. doi: 10.1038/s41598-024-66620-x (PMC11233627; doi:10.1038/s41598-024-66620-x)
Supplement: Supplementary file 1 — Supplementary Information 1. [file 41598_2024_66620_MOESM1_ESM.docx]

**Supplementary figures captions:**

Fig S1 Wood decay visualization after 13 and 20 days of inoculation of *F. mediterranea* on the sawdust of three cultivars of grapevine CS, UB and MT.

Fig S2 Ugni blanc sawdust inoculated or not with bacterial strains and/ or *F. mediterranea*

**Supplementary tables captions:**

Table S1 RAST annotation of genome of strain S231-2

Table S2 RAST annotation of genome of strain S293

Table S3 Genes in subsystems detected in S231-2 but not in S293

Table S4 Genes in subsystems detected in S293 but not in S231-2

Table S5 Eggnog annotation of genome of strain S231-2

Table S6 Eggnog annotation of genome of strain S293
