# Supplementary material for: Two Paenibacillus spp. strains promote grapevine wood degradation by the fungus Fomitiporia mediterranea: from degradation experiments to genome analyses
Source: Sci Rep. 2024 Jul 9;14:15779. doi: 10.1038/s41598-024-66620-x (PMC11233627; doi:10.1038/s41598-024-66620-x)
Supplement: Supplementary file 2 — Supplementary Information 2. [file 41598_2024_66620_MOESM2_ESM.docx]

**
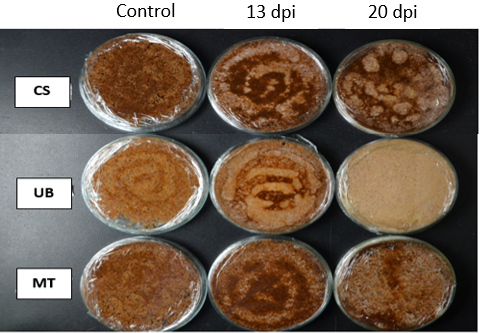
**

Fig S1 : Wood decay visualization after 13 and 20 days of inoculation of *F. mediterranea* on the sawdust of three cultivars of grapevine CS, UB and MT.


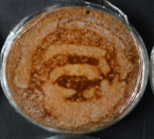

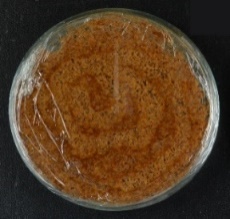


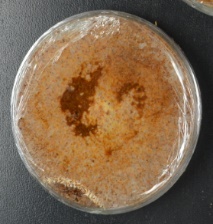


Fm

Control


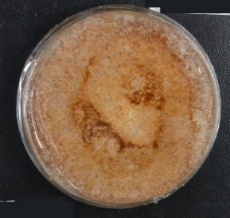

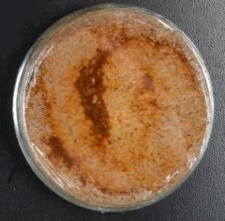


Fm/S231

Fm/S293

Fm/S293/S231
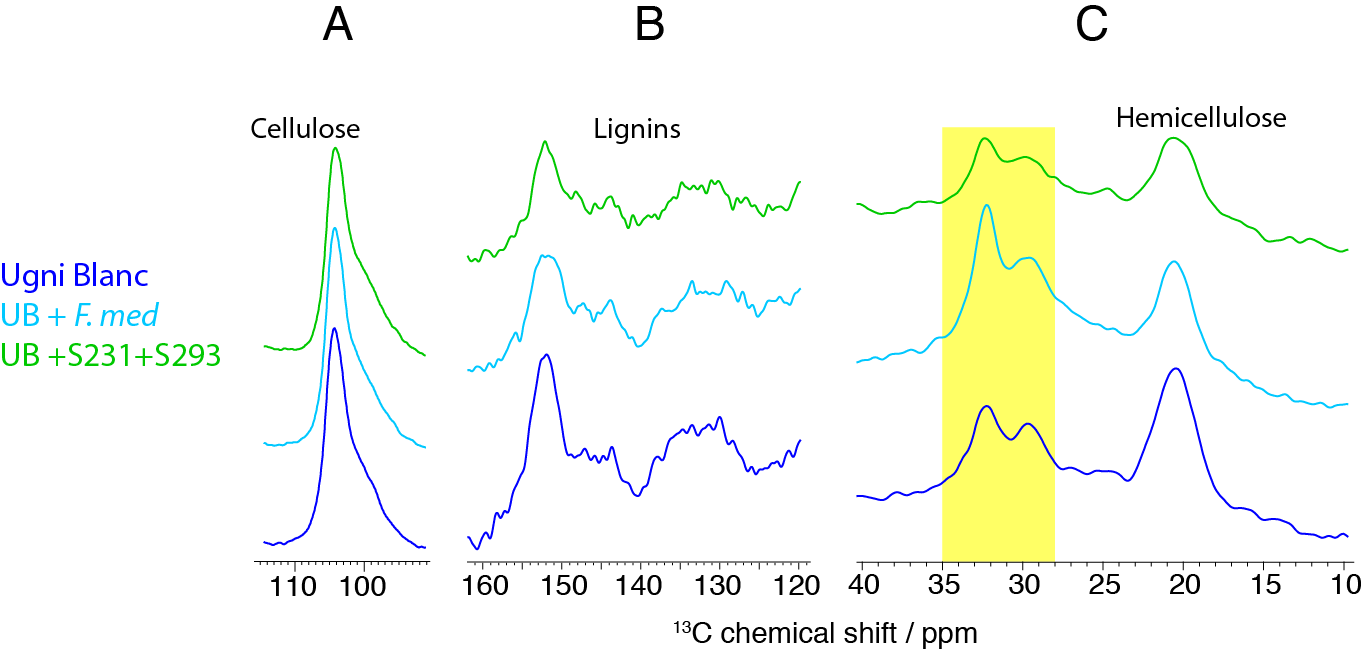


Fig S2 :Ugni blanc sawdust inoculated or not with bacterial strains and/ or *F. mediterranea*
